# Bifid Mandibular Condyle as the Hidden Cause for Temporomandibular Joint Disorder

**DOI:** 10.7759/cureus.17609

**Published:** 2021-08-31

**Authors:** Rajashri R, Senthilnathan Periasamy, Santhosh P Kumar

**Affiliations:** 1 Oral and Maxillofacial Surgery, Saveetha Dental College and Hospital, Chennai, IND

**Keywords:** bifid mandibular condyle, temporomandibular disorders, tmj pain, tmj disc degeneration, congenital disorder, surgical case report

## Abstract

Bifid mandibular condyle is a rare occurrence, more frequently unilateral. The etiology of this condition is controversial. Bifid mandibular condyles of developmental origin are mostly asymptomatic and discovered incidentally through imaging. Here, we report a 38-year-old male patient, previously in good health, presented with progressive pain in his right temporomandibular joint and restricted joint movements. MRI of the bilateral temporomandibular joints revealed mild degenerative disc on the right side and bifid mandibular condyle on the left side. Conservative treatment comprising a series of soft, medium, and hard splint therapy in combination with analgesics showed symptomatic improvement initially but did not improve the condition in the long term. He subsequently underwent arthrocentesis of the right temporomandibular joint and perceived a good clinical improvement until he developed progressive pain in the left temporomandibular joint and radiating to the left side of the face. He underwent partial condylectomy and discopexy following which all of his symptoms improved; which drives us to question if bifid mandibular condyle is the hidden cause for bilateral temporomandibular joint pain.

## Introduction

Bifid mandibular condyle (BMC) is an extremely rare anatomic variation of controversial etiology. It is a developmental abnormality but also associated with trauma, infection, irradiation, vascular anomalies, abnormal muscle pull, condylar fractures, condylectomy, and nutritional, endocrinal, genetic, or teratogenic factors [1]. Most bifid condyles are asymptomatic and discovered only as an incidental finding during routine radiographic examination using orthopantomograph [2]. However, with the use of advanced imaging modalities like computed tomography (CT), cone-beam computed tomography (CBCT), and magnetic resonance imaging (MRI), there is an increase in the incidences, accounting for about 0.31% to 1.82% of cases [3]. Symptomatic cases present with one or more signs like pain, swelling, noise, hypomobility, joint block, deflection, joint luxation, or even ankylosis. Clinical manifestations can be sudden or gradual pain, but most often precipitated by trauma to the region [4,5]. In this article, we present a rare case of unilateral bifid mandibular condyle causing pain in the bilateral temporomandibular joints and the management of the condition.

## Case presentation

**Figure 1:**
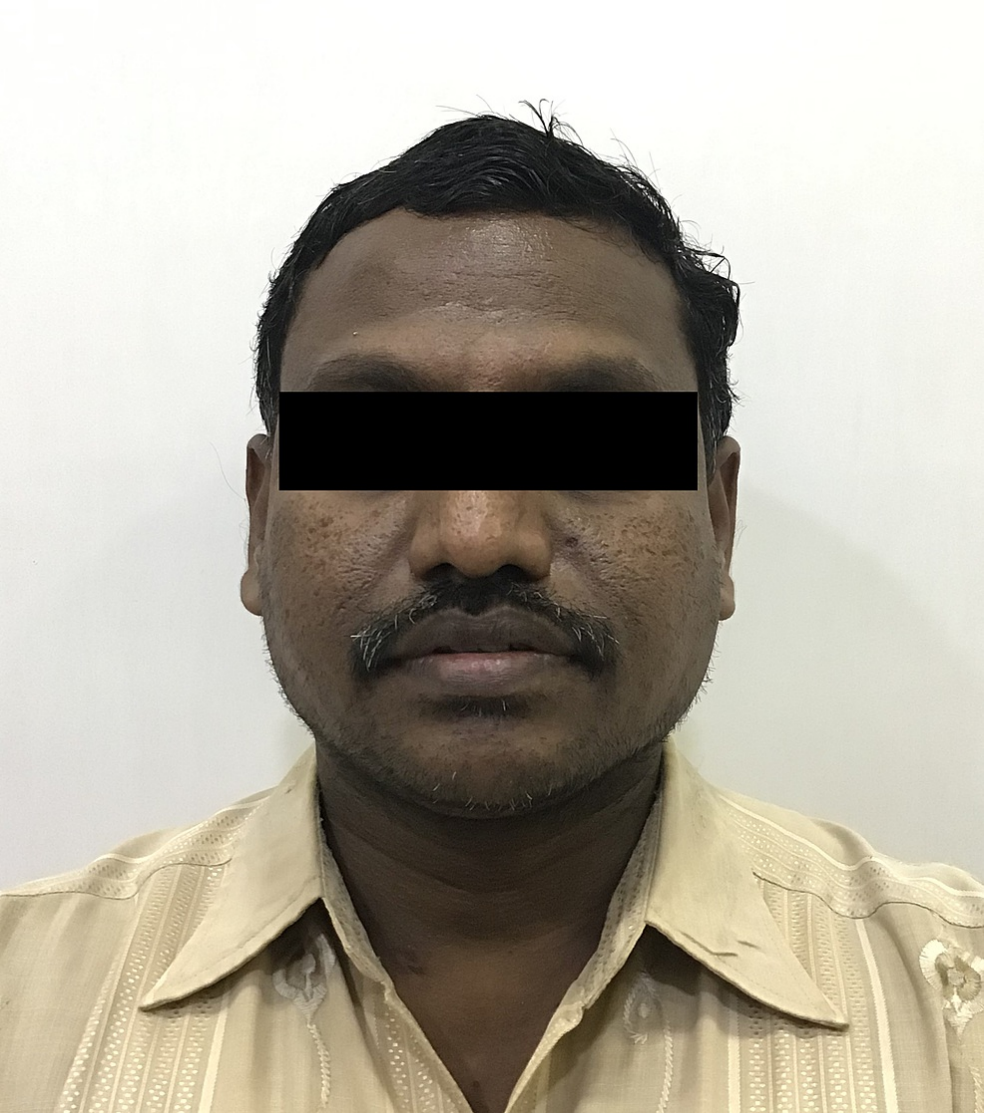
Pre-operative frontal picture

We conducted this study under the ethical standards of the Institutional Review Board of our institution. A 38-year-old moderately built male patient presented with progressive pain in his left temporomandibular joint as well as radiating pain on the left side of the face (Figure 1).

**Figure 2:**
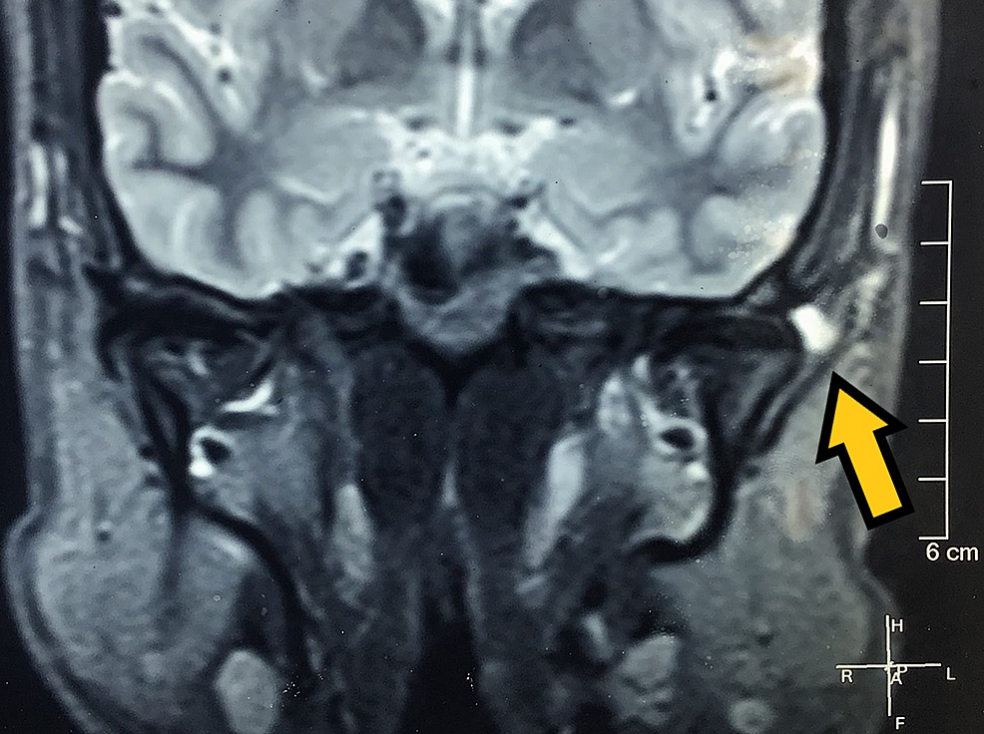
Pre-treatment MRI (coronal section) showing disc and left condyle (pointed by the arrow) in closed-mouth position.

**Figure 3:**
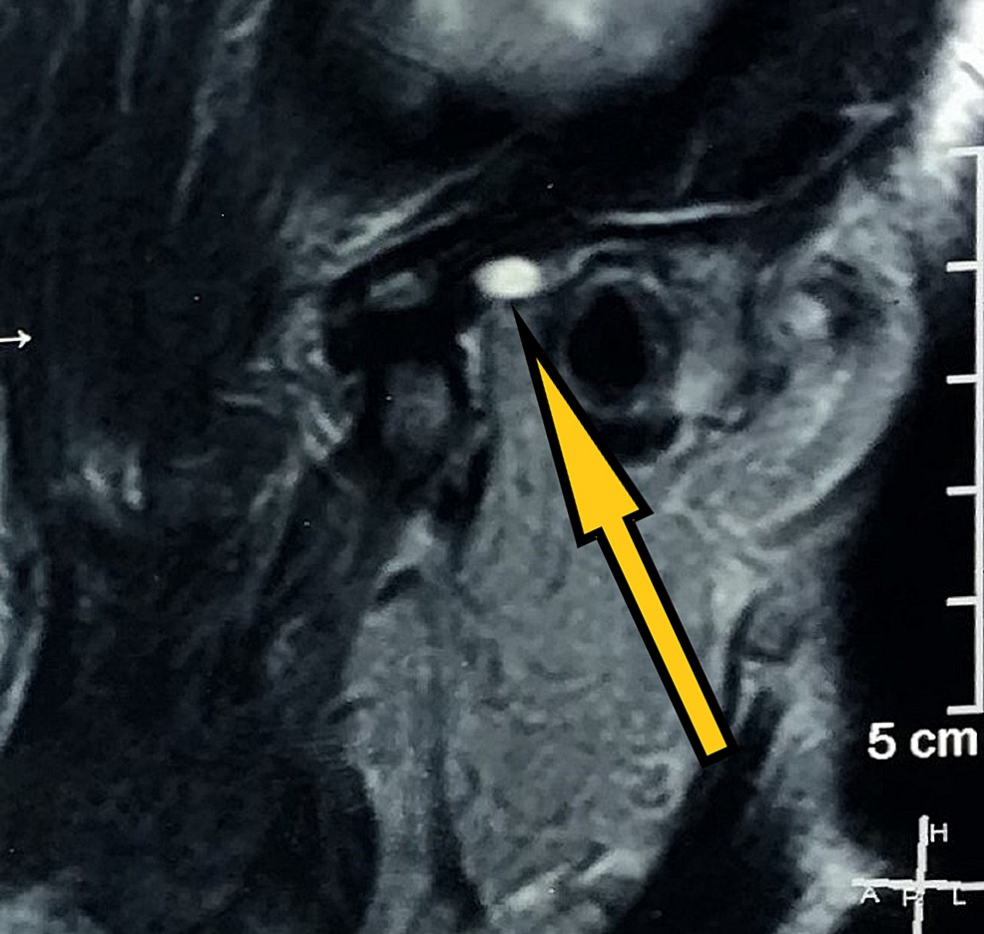
Pre-treatment MRI (sagittal section) showing disc and left condyle in open-mouth position.

History revealed symptoms first presented as pain in the right temporomandibular joint and impaired mouth opening during speech and mastication. He also gave a history of clicking sounds in the left temporomandibular joint since childhood with no pain. Upon initial physical examination, we found the patient to have no tenderness on palpation in his left temporomandibular joint. He experienced tenderness on the right side with restricted mouth opening. His previous MRI of the bilateral temporomandibular joint showed signs of mild degenerative changes of the bilateral articular disk with reduced translation on the right side and bifid left mandibular condyle with a small cyst next to the left temporomandibular joint (Figures 2, 3).

**Figure 4:**
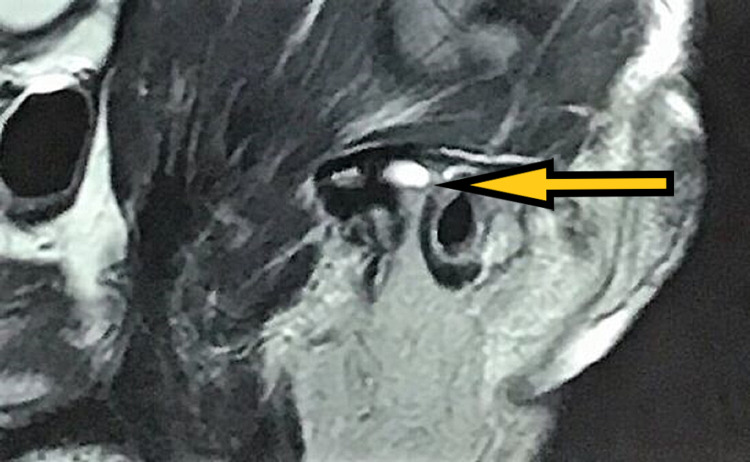
Post-arthrocentesis MRI (sagittal section) showing disc and left condyle in closed-mouth position.

**Figure 5:**
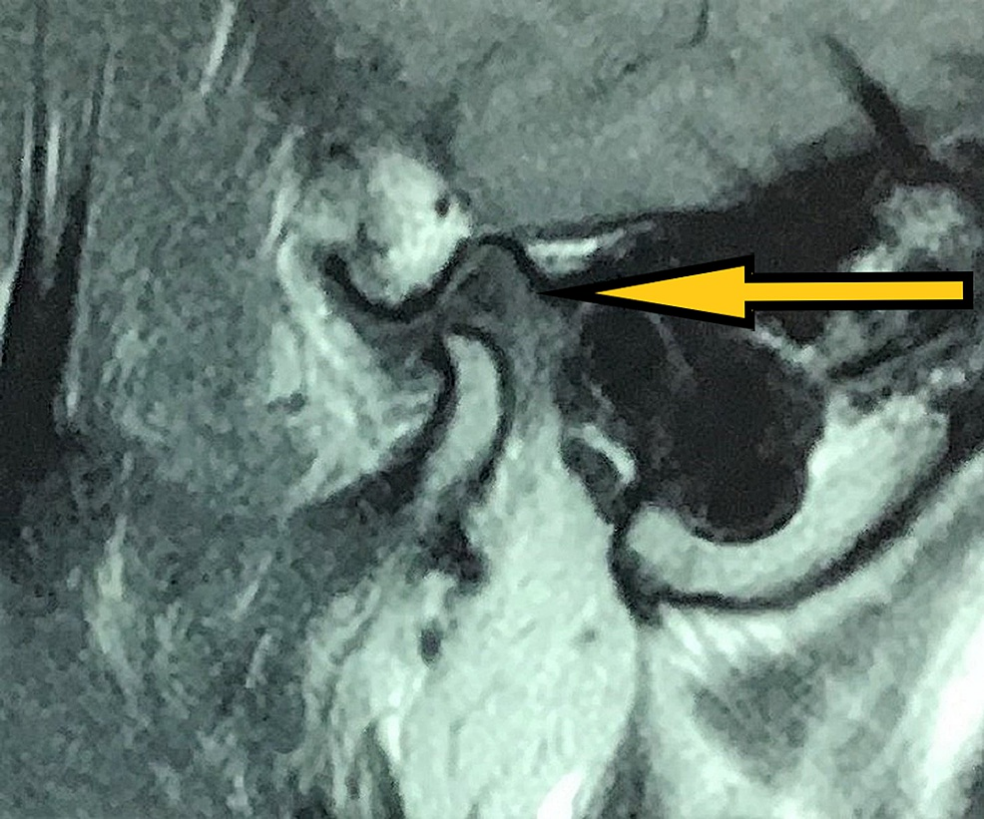
Post-arthrocentesis MRI (sagittal section) showing disc and the right condyle in open-mouth position.

The muscles of mastication did not show any reproducible pain, and erythema or edema was absent bilaterally. He endorsed decreased pain in the right temporomandibular joint following a series of soft, moderate, and hard splint therapy in sequence for a period of four months each on average and in combination with analgesics for two years. However, his pain persisted on the right side, and he discontinued the use of splints. As his symptoms worsened over one year, he underwent arthrocentesis under local anaesthesia for the right temporomandibular joint using the double cannula technique. Ringers lactate was used to flush the joint space, followed by viscosupplementation with sodium hyaluronidase. He resumed wearing hard splints and was then asymptomatic for 18 months, following which he developed progressive pain in the left temporomandibular joint that radiated over his left ear. MRI revealed impingement of anterior or intermediate disc band caused by the left bifid condyle with minimal anterior translation, and there was evidence of mild degenerative arthropathy in his right temporomandibular joint (Figures 4, 5).

**Figure 6:**
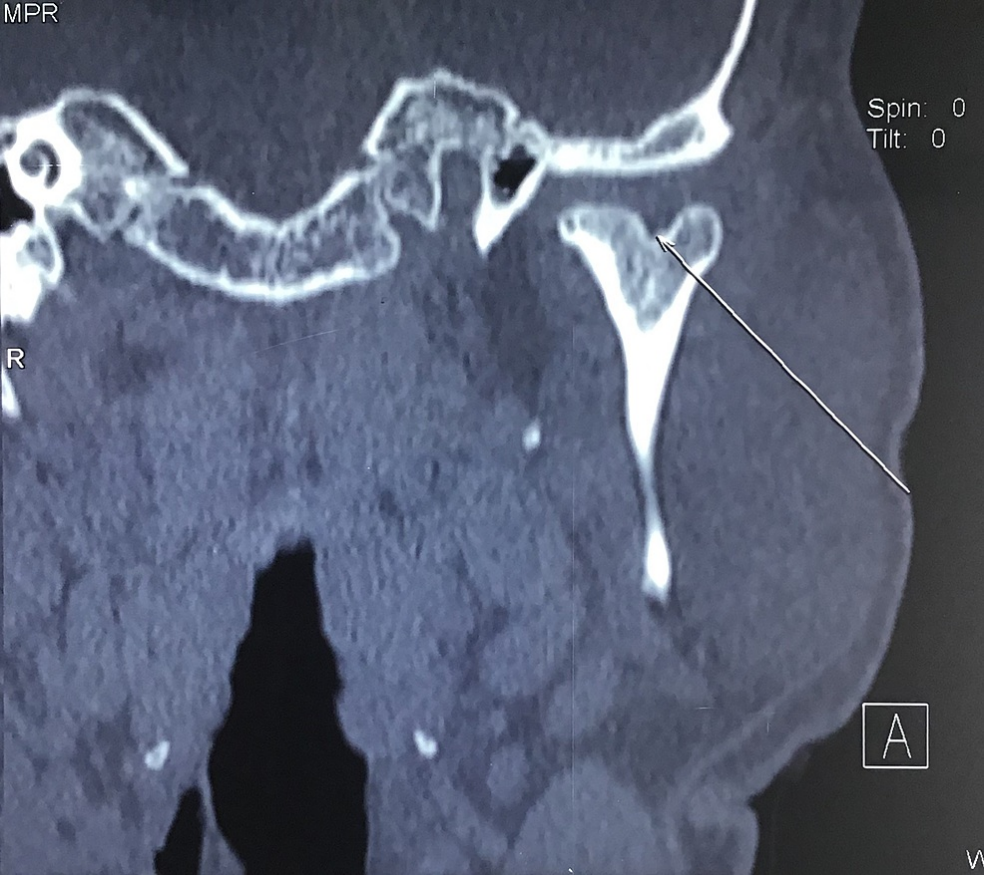
Pre-operative CT (coronal section) depicting left bifid condyle.

CT was taken to confirm the presence of bifid mandibular condyle and study its morphology (Figure 6).

**Figure 7:**
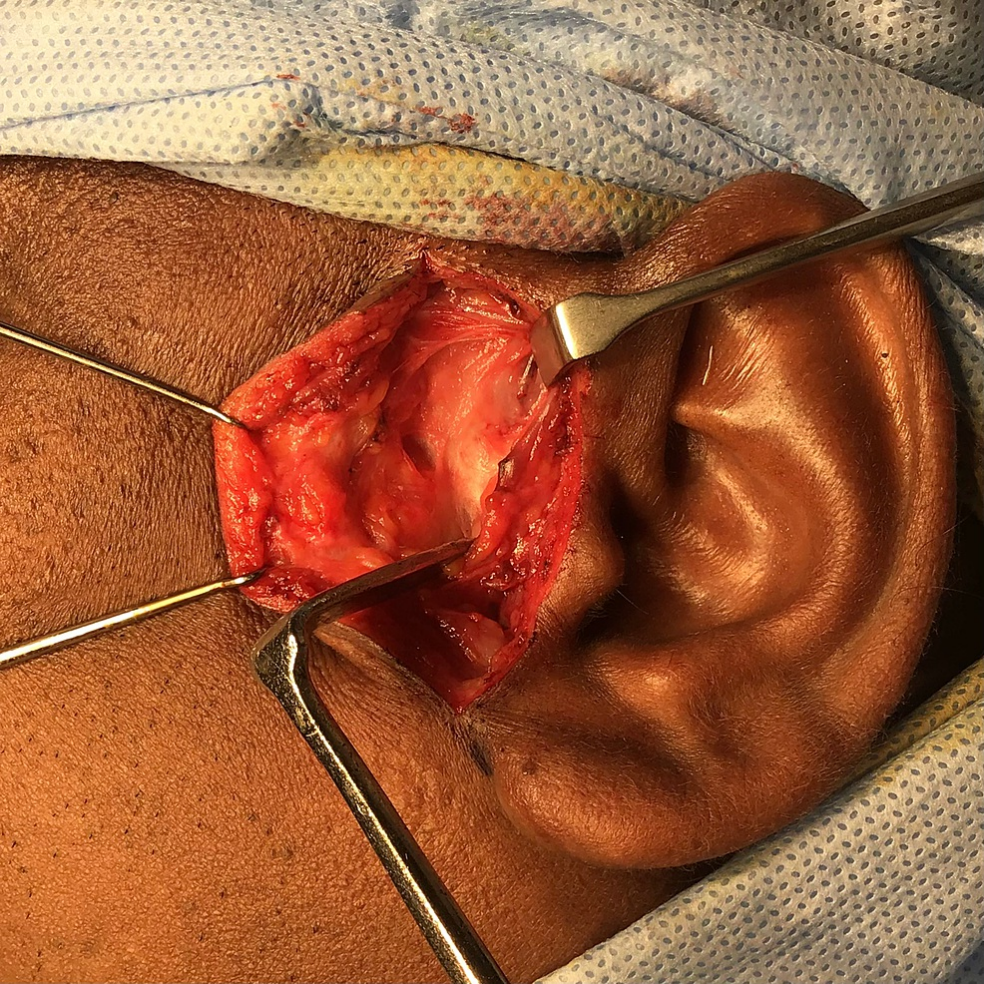
Intra-operative picture showing incision and dissection to reach the fascia.

Based on history, clinical features, and radiographic investigations, high condylectomy of the lateral pole of bifid condyle and discopexy was planned under general anaesthesia. Using the preauricular incision, dissection was carried to reach the temporalis fascia and incised until we saw the temporalis muscle. We cut through the fascia and periosteum over the root of the zygoma near the post glenoid tubercle (Figure 7).

**Figure 8:**
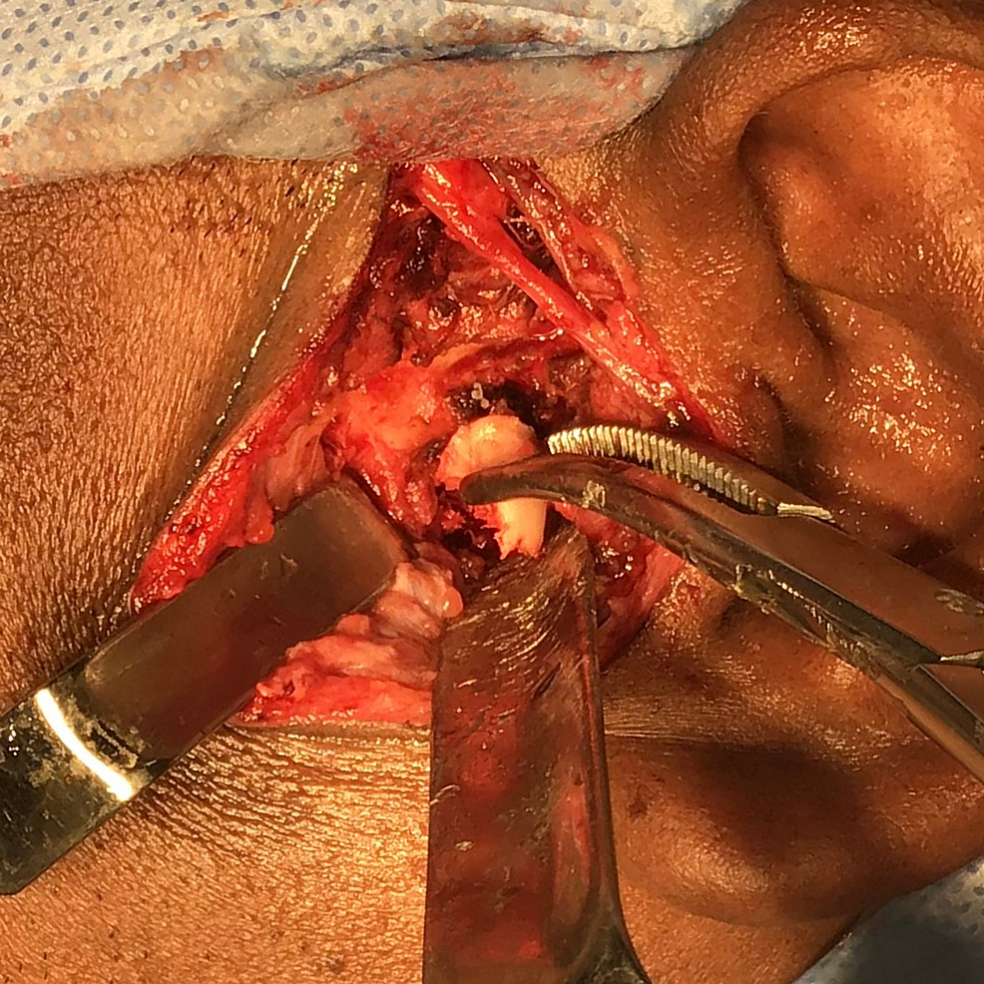
Intra-operative picture showing left lateral pole of the condyle removed in toto.

The incision was continuous and anterior and superior to the tragus and subperiosteal dissection was done forwards off the arch. The joint capsule was identified and incised to divide the posterior and superior attachments and dissected further to expose the disc, condylar head and neck. Retraction was done to perform osteotomy in the supero-inferior direction of the condyle, dividing the lateral portion of the bifid condyle through the notch. A final tap using the osteotome completed the division (Figure 8).

**Figure 9:**
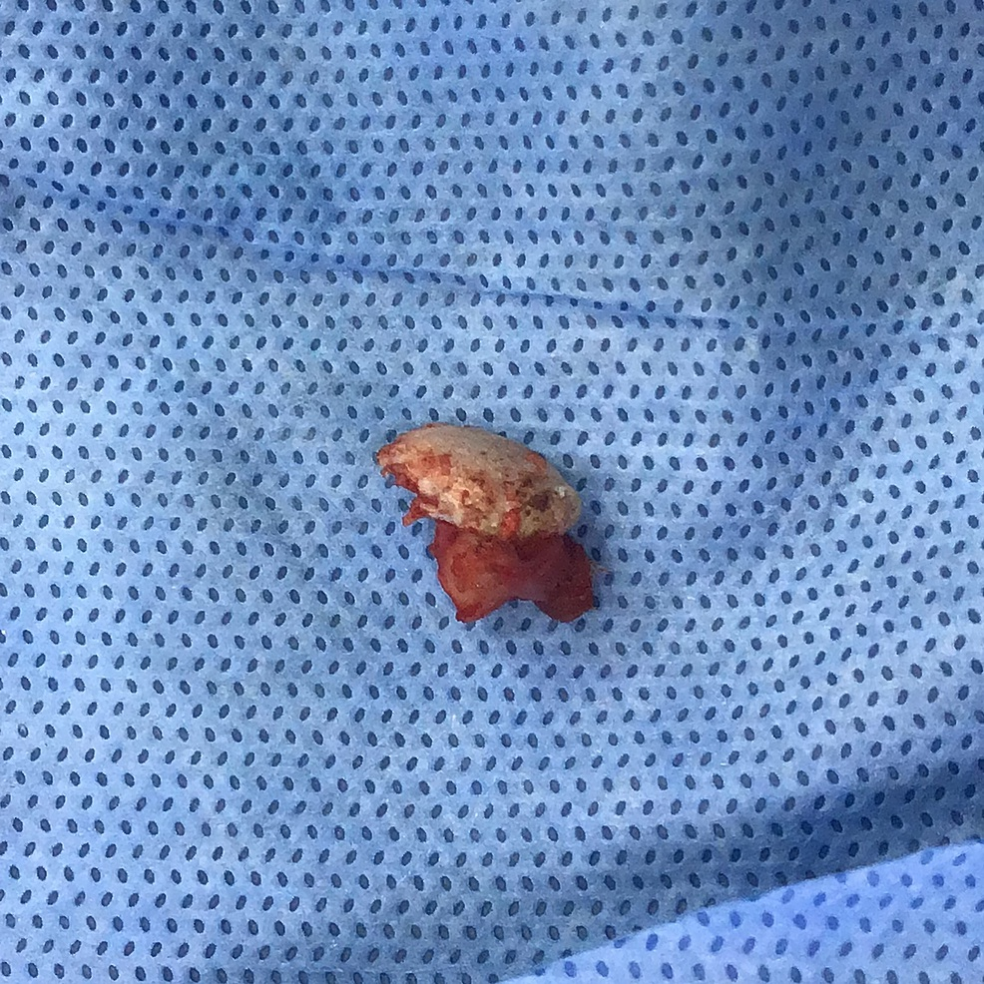
Excised left lateral pole of the condyle (Specimen)

The condylar mass was removed in toto and the specimen was sent for histopathological interpretation (Figure 9).

The bony margins were smoothened, and bone wax was placed. Discopexy was done by suturing the disc in the joint cavity using 4-0 ethilon (Ethicon Inc., New Jersey, USA). After achieving hemostasis, closure was done in layers using 3-0 vicryl (Ethicon Inc., New Jersey, USA) and 4-0 ethilon.

**Figure 10:**
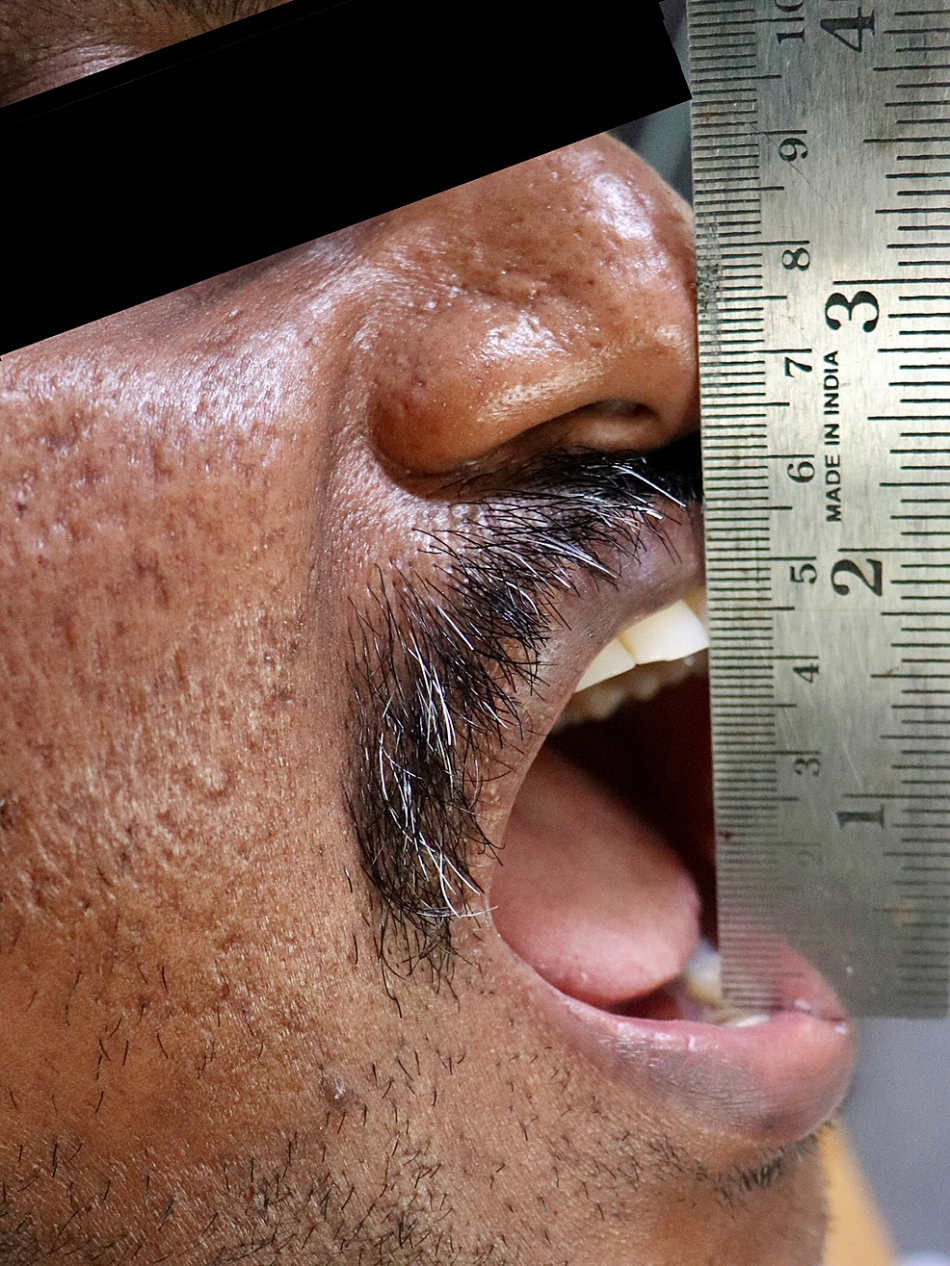
Post-operative maximum inter incisal opening of 42mm without symptoms depicting adequate joint functions.

The histopathological report suggested normal condylar bone. On postoperative three-month follow-up, improvement in mouth opening and decreased pain over the temporomandibular joint was observed clinically (Figure 10).

**Figure 11:**
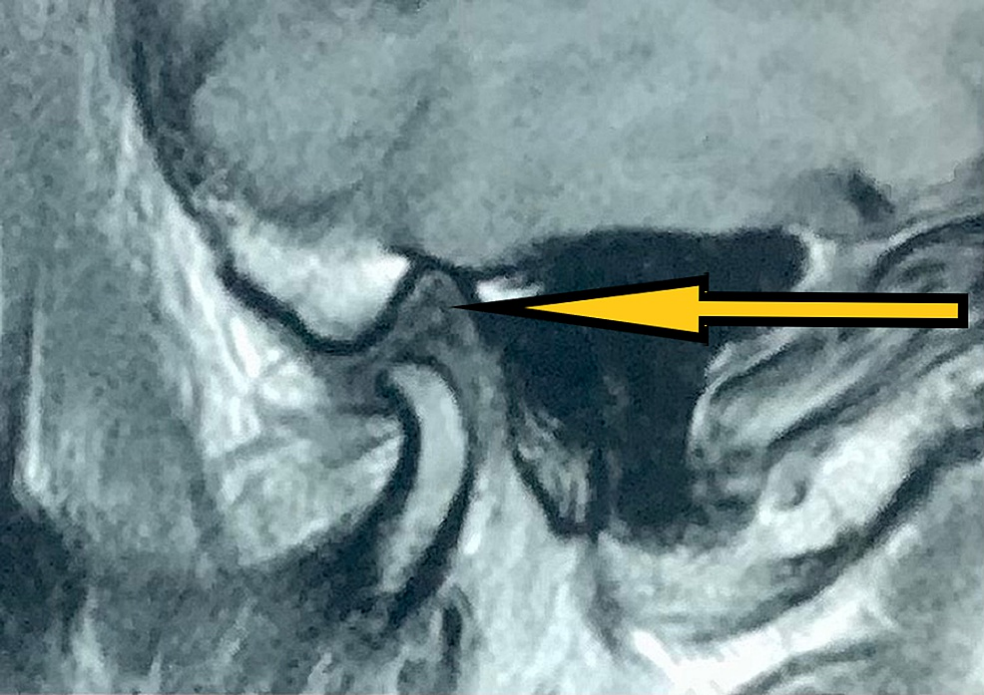
Post-surgical MRI (sagittal section) at nine months follow-up showing normal translation and position of the disc in open mouth position on the right side temporomandibular joint.

During the nine-month follow-up, the patient had satisfactory mouth opening and temporomandibular joint functions. His postoperative MRI at nine months follow-up on the right side revealed a contoured articular disc and position in closed-mouth position. Normal translation and position of the disc in open mouth position. No obvious deformity of the articular disc was visualised. No fluid was visualised in the joint. No bone erosions or synovial hypertrophy were visualised. Likely marginal osteophyte is seen in the right condyle (Figure 11).

**Figure 12:**
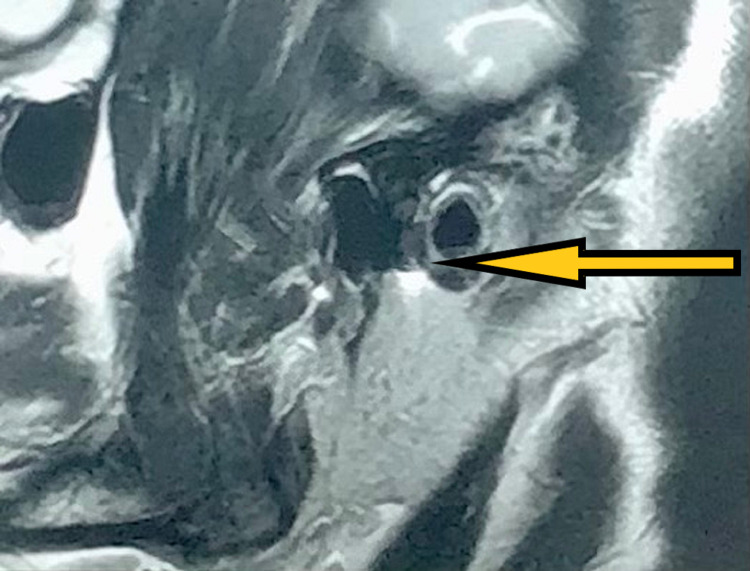
Post-surgical MRI (sagittal section) at nine months follow-up showing the post-surgical cortical defect in the left hemicondyle.

On the left side, there is a partial post-surgical defect in the hemicondyle. No evidence of hyperintensity or edema was observed. There is no evidence of abscess or fluid collection suggesting successful management of the temporomandibular disorder (Figure 12).

## Discussion

Bifid mandibular condyle is an extremely rare occurrence [6]. It was first described as a condylar split or groove of variable depth in 1941 [7]. However, the condylar split can range from a shallow groove to the formation of two condylar heads, oriented either mediolaterally or anteroposteriorly [8]. The origin of the bifid mandibular condyle is highly controversial and many theories exist [9]. Some authors consider it to be a developmental abnormality where they are asymptomatic and detected only through a routine radiographic examination using an orthopantomogram (OPG). With the use of advanced imaging modalities such as computed tomography (CT), cone-beam computed tomography (CBCT), and magnetic resonance imaging (MRI), there has been an increase in the number of cases reported with an actual prevalence of about 0.31% to 1.82% [3]. For diagnosis, MRI and CT are the most useful tools to identify bifid mandibular condyle. In some patients, identifying bifid condyle using OPG is futile because of the masking of the two poles of the bifid condyle and the inability to appreciate the anatomy through OPG [10].

Hrdlicka tried to explain the origin through the obstructed blood supply to the condyle during its development, resulting in the division to the condyle [7]. Blackwood suggested vascularized fibrous septa, by examining the condylar cartilage of 10 human skulls, which disappeared two years after birth [11]. Thus, it was concluded that the septum along with a blood blockage influences ossification, resulting in a BMC. Poswillo et al. postulated that changes in the position of disc or form stimulate the formation of intra-articular septa across the joint space [12]. Correlating the conclusions by these authors on the origin of BMC with our patient's history of clicking sounds in the left temporomandibular joint and no history of trauma or pain in his temporomandibular joints, we hypothesized that the bifid condyle is of congenital origin.

It is interesting to note from the recent systematic review that 17.2% of cases have reported clicking sounds, 18.1% have arthralgia and 22.7% have hypomobility. The treatment options were active monitoring, use of the occlusal splint, and use of analgesics. They considered joint surgery to be the last resort in cases that are resistant to conservative treatment, accounting for 15.7% [3]. 

We managed conservatively initially and his symptoms disappeared on the right side. However, his symptoms worsened on the left side, hampering his daily activities. Surgical excision of the lateral pole of the bifid condyle offered pain relief according to the patient. Considering the overall outcomes of conservative management and surgery, both joints are in good function. However, it is unclear if bifid condyle could have been the initiator of the temporomandibular joint pain on both sides as the etiology of degenerative joint disease is also of unknown origin largely. There is potential scope for more research. More studies need to be conducted to decipher the etiology and the relationship of bifid mandibular condyle with temporomandibular joint disorders.

## Conclusions

We present a rare case of asymptomatic left bifid mandibular condyle with degenerative articular disk on the right side in a healthy 38-year-old male. Conservative management showed clinical improvement on four-year follow-up on the right side, however, his symptoms progressively worsened on the left side. We performed condylectomy on the left side and the patient is currently asymptomatic for nine months. Since the etiologies of both bifid Condyle and disc degeneration are unknown, there is a high ambiguity in deciphering the cause of the disease. There is a possibility that bifid condyle could also be a factor despite the age-related factors in causing disc degeneration. Based on the outcomes, we believe that there is a necessity to establish the relationship between bifid mandibular condyle and temporomandibular disorders.
